# Characteristics and impact of pain from root-filled teeth. A practice-based cross-sectional study comparing painful teeth with and without signs of inflammatory dental disease

**DOI:** 10.22514/jofph.2024.007

**Published:** 2024-03-12

**Authors:** Jakob Jonsson Sjögren, Thomas Kvist, Thomas List, Alf Eliasson, EndoReCo, Maria Pigg

**Affiliations:** ^1^Dental Research Department, Public Dental Health Service, 701 16 Örebro, Sweden; ^2^Department of Endodontics, Faculty of Odontology, Malmö University, 205 06 Malmö, Sweden; ^3^Department of Endodontology, Institute of Odontology at the Sahlgrenska Academy, University of Gothenburg, 405 30 Gothenburg, Sweden; ^4^Department of Orofacial Pain and Jaw Function, Faculty of Odontology, Malmö University, 205 06 Malmö, Sweden; ^5^School of Health and Medical Sciences, Örebro University, 701 82 Örebro, Sweden

**Keywords:** Dentistry/diagnosis, Dentistry/epidemiology, Endodontics, Facial pain, Pain, Root canal therapy

## Abstract

To compare pain characteristics, impact of pain and characteristics of patients with painful root-filled teeth with and without signs of inflammatory dental disease. This cross-sectional study was performed in the Public Dental Health services, Region Örebro County, Sweden. Adult patients with ≥1 root-filled tooth identified at their regular check-up were included and assigned to one of two groups; those with ≥1 sign of inflammatory dental disease (DD+) and those without any such sign (DD−). Patients/teeth were compared regarding pain characteristics (intensity, frequency, duration, quality and provoking factors), impact of pain (medication intake, impact on life) and patient characteristics as background factors (general health, other bodily and orofacial pain). Statistics included descriptive data (frequency tables) and group comparisons (Chi-square, Fisher’s Exact and Mann-Whitney U-tests). The DD+ group included 27 participants (30 teeth) and the DD− group 22 participants (23 teeth). On average, pain intensity was mild, the frequency most often recurrent, and the impact was low. Average pain duration since onset exceeded 2 years in both groups. The only observed between-group differences were average pain intensity; 3.1 (0–10 Numerical Rating Scale (NRS)) in DD− group compared to 1.6 for DD+ (*p* = 0.030), and tenderness to apical palpation; only reported in the DD+ group. The similarities in clinical presentation between the two groups underscore the difficulties in correctly distinguishing between pain of odontogenic and non-odontogenic origin in root-filled teeth with a standard clinical investigation. Additional diagnostic methods need to be investigated for their ability to differentiate between tooth pain or discomfort of different origins.

## 1. Introduction

In investigations of painful root-filled teeth, 9.6%–12% of individuals report pain associated with at least one of their root-filled teeth [1, 2, 3]. Reasons for persistent pain attributed to disease or conditions affecting teeth include apical periodontitis (AP), vertical root fracture [4, 5], traumatic occlusion [6] and marginal periodontitis [7].

But root-filled teeth without signs of inflammatory disease can also be painful. In a practice-based cross-sectional study in Sweden no significant association between pain or discomfort from root-filled teeth and apical radiolucency was reported when controlling for other factors, and only 41.9% of the painful teeth exhibited apical radiolucency [3]. There are several different possible origins for tooth pain that cannot be attributed to a dental problem, which may in part explain the observed variation [3]. The prevalence of non-odontogenic tooth pain 1–6 years after root canal treatment has been estimated to 3.1–3.4% [4, 8]. Possible pain origins include referred pain from temporomandibular disorders (TMDs) [9, 10], sinusitis [11], and neuropathic pain secondary to nerve injury [12, 13], neurovascular pain/headache [14] but the origin can also be unknown, *i.e.*, persistent idiopathic dentoalveolar pain [15]. From a disease or disorder perspective, odontogenic and non-odontogenic pain likely represent completely different pain mechanisms, and it seems reasonable to assume that with different pain origins, the clinical presentation would also differ. A clinical follow-up (n = 19) six months after root canal treatment (RCT) reported differences in pain intensity and ability to localize the pain between individuals with odontogenic and non-odontogenic pain origins [10], but other possible differences in pain characteristics have not been extensively studied.

The clinical differentiation between painful conditions is not straightforward. Signs and symptoms overlap, and it can be difficult to make a correct diagnosis [16]. It is not known if odontogenic (*e.g.*, endodontic) and non-odontogenic tooth pain differ in frequency, quality, intensity, or impact on the patient’s life. It is also unknown if background factors on the individual level, such as systemic health, chronic bodily pain or other orofacial pain are associated with persistent pain from root-filled teeth, but it is well known that comorbidity exists between chronic pain conditions [17, 18].

Comparing participants and their painful root-filled teeth with and without clinical signs of dental disease on both tooth level and individual level may lead to a better understanding of what characterizes tooth pain of different origins. Furthermore, for individuals with persistent pain, the consequences of pain are often more problematic than the pain itself and may require more complex management [19]. Increased knowledge could contribute to clinical management by indicating strategies for adequate diagnostics as well as management of root-filled teeth displaying symptoms.

The aim of this study was to compare painful root-filled teeth that display signs of dental inflammatory disease (DD+) and teeth without such signs (DD−) concerning (i) pain characteristics, (ii) impact of pain and (iii) patient characteristics (background factors).

The underlying hypothesis, based on the reported difficulties to distinguish clinically between tooth pain of dental and non-dental origin [16, 20, 21], was that no differences between the groups would be found.

## 2. Material and methods

The study conforms to the STROBE guidelines for observational studies [22] and to the guidelines for Preferred Reporting items for Observational studies in Endodontics (PROBE) [23].

### 2.1 Study design and participants

This is a cross-sectional study using data collected during the year 2015 in Örebro County, Sweden. All adult patients with at least one root-filled tooth who were scheduled for routine examination in April 2015 were eligible and invited to participate. The recruitment and examinations were performed within the 23 clinics of the Public Dental Service in Örebro County. The patients were thus examined by their regular dentist, who then provided the data to the study coordinator (JJS). For further details on design, see Jonsson Sjögren *et al*. [3] (2019).

### 2.2 Enrolment and data collection

If a patient agreed to participate, all their root-filled teeth were included in the data collection in 2015. The forms of informed consent are stored in a locked room of the Public Dental Service in Örebro County. Participants received no monetary compensation for study participation.

### 2.3 Inclusion in the present study

The individuals with one or more painful root-filled tooth identified in the data collection in 2015 were included in the analyses of this study. The definition of “painful” was the subjective experience reported by the patients.

The data collection included the following:

Clinical assessment of all included teeth to identify clear signs of infection/inflammation: presence of swelling, presence of sinus tract and greatest probing depth at the tooth.

The quality of the coronal restoration was rated as either good or poor (secondary caries, defective temporary restoration, permanent restoration with insufficient marginal integrity or no restoration). Tenderness to percussion and to apical palpation indicating hyperalgesia or allodynia were also noted.

• Radiographic examination of the root-filled tooth by intraoral periapical images with all apices clearly visible. It sufficed with one image if the first radiograph, exposed with an orthoradial projection, showed a clearly identifiable radiolucency around any apex. If the first image however appeared to show a normal periodontal ligament, *i.e.*, healthy conditions, a second periapical radiograph was exposed, with a 10–15 degrees overaxial or a mesial or distal eccentric projection to improve detection of periapical radiolucency [24].

Clinical and radiographic examination findings were combined to form two groups based on whether the examination revealed signs of dental disease or not, see “Definition of groups” below.

• An interviewer-assisted questionnaire comprising questions regarding general health (diabetes, cardiovascular disease, gastrointestinal disorder, rheumatic disease, neurological disease, psychiatric disorder). Individuals were categorized as either “healthy” or “with one or more general health concerns”.

• Screening questions for TMD (3Q/TMD) [25]. The two questions specifically related to pain were used; the third question concerns locking of the jaw and was not considered relevant in this context.

Q1: Do you have pain in your temple, face, jaw or jaw joint once a week or more? (YES/NO)

Q2: Do you have pain once a week or more when you open your mouth or chew? (YES/NO)

• Occurrence of other chronic pain conditions, lasting at least 3 months [26] and persistent in character: “During the last three months, have you experienced pain in more than one place in the body four days per week or more often? (YES/NO)” [27].

The patients of this study all answered YES to the question if they had experienced any pain or discomfort (such as tenderness or swelling) related to the root-filled tooth during the last 3 months. They then completed a second questionnaire, one for each painful root-filled tooth, comprising questions regarding pain characteristics and consequences of pain:

(i) Pain intensity: current pain, average pain during the last month, and worst pain during the last month (on an 11-point numeric rating scale (NRS) ranging from 0 = no pain to 10 = pain as bad as could be).

(ii) Pain duration since onset: in months since the pain was first experienced.

(iii) Pain frequency: if the pain is continuous, recurrent or occasional.

(iv) Provoking factors: if the pain occurs spontaneously or when using, irritating or provoking the tooth.

(v) Pain persistence: if the pain from the tooth has lasted at least 8 hours a day and at least 15 days per month the last 3 months [28].

(vi) Pain quality: rated according to the short-form McGill Pain Questionnaire (SF-MPQ), in which 15 descriptive words of the pain are rated on a 4-point scale (0 = none, 1 = mild, 2 = moderate and 3 = severe). Eleven words describe the sensory part of the pain experience and four words describe the affective part [29, 30].

(vii) Pain intensity and pain-related disability: assessed using the compound measure Graded Chronic Pain Severity Scale (GCPS) [31, 32, 33]. The scale was modified to measure pain in a 1-month perspective [34]. The different measures of the GCPS were calculated; Characteristic Pain Intensity (CPI), disability points for number of days with interference, interference score and disability points for the interference score. These, in turn were used to calculate the compound measure Chronic Pain Grade, (0 = none, I = low intensity pain, with none-to-low pain-related disability, IIa = High intensity pain, without pain-related disability, IIb = High intensity pain, with low pain-related disability, III = Moderately limiting and IV = Severely limiting).

Characteristic pain intensity: On an 11-point Numeric Rating Scale (0–10 NRS) where zero represents “no pain” and 10 “pain as bad as could be”, participants rated the pain at the time of the examination, the worst pain experienced the past month, and the average pain experienced the past month. The mean of the three NRS scores was multiplied by ten to form the CPI, modified from Ohrbach (2010) [32].

Pain-related disability: The participants also stated the number of days the last month the pain was experienced, the number of days the pain had caused them to abstain from normal activities, and the degree of limitation that the pain caused for each of the three domains daily activities, recreational activities, and ability to work. Each was rated on the 11-point numeric rating scale (0–10 NRS), with 0 representing no limitation at all and 10 representing “unable to perform any activities”. The mean of the three NRS scores was multiplied by ten to form the disability score.

Disability points: The sum of the points of disability days and the disability score added together.

(viii) Present or recent intake of, *e.g.*, analgesics, antibiotics, herbal remedies for the pain.

For further details on examination and data collection, see Jonsson Sjögren *et al*. [3] (2019).

### 2.4 Definitions of groups

Clinical and radiographic findings were combined when determining if a tooth was considered affected with dental inflammatory disease or not. Regarding the clinical findings, presence of swelling, a sinus tract and deep periodontal pocket/-s (≥6 mm) were considered as certain signs of an inflammatory condition affecting the surrounding tissues of the tooth. In contrast, we did not regard tenderness to percussion and to apical palpation as definite signs of inflammatory dental disease, since hyperalgesia and allodynia (gain in sensory function) often occur also due to pain of other origin [35, 36, 37].

Radiographic findings included presence/absence of a periapical radiolucency. Three specialists in Oral Radiology reviewed the radiographs independently of each other under optimal conditions without access to information about symptoms and clinical status. The observers noted if the apical condition was normal; if there was a widened periodontal ligament space ≤1 mm or if there was an apical radiolucency >1 mm wide. Given that generally, interobserver agreement on assessment of apical status is low for intraoral radiography [38], a majority principle in rating the apical status was followed, *i.e.*, if two observers agreed on apical radiolucency the tooth was noted as having apical radiolucency. To obtain a strict definition of apical radiolucency and thereby avoid overestimating the presence of periapical disease, the ratings “normal apical condition” and “widened periodontal ligament space” were merged into one group, representing absence of radiolucency.

The two study groups were defined as follows:

Dental disease positive (DD+), a painful root-filled tooth with at least one positive finding of swelling, sinus tract, pocket depth ≥6 mm, or apical radiolucency.

Dental disease negative (DD−), a painful root-filled tooth without any of the findings swelling, sinus tract, pocket depth ≥6 mm, or apical radiolucency.

### 2.5 Statistical methods

A power analysis was performed. Data from 48 patients was available, 36 who had signs of dental disease and 12 without such signs. The basis for the analysis was pain intensity, and a difference of 3 units on the 0–10 NRS was assumed as clinically relevant [39]. A standard deviation (SD) of 1.8 was assumed from a study of similar patients [2]. The effect size was 1.67. With a 5% risk of type I error and a power of 80% at least six cases per pain group were needed to identify a statistically significant difference.

Data were entered in Excel® sheets (2401 Build 16.0.17231.20170, Microsoft Corporation, Redmond, WA, USA) and transferred to IBM SPSS version 27 (IBM, Armonk, NY, USA) for statistical analysis. Descriptive statistics was used for patient characteristics as well as pain characteristics. For comparisons between groups, Chi-square test and Fisher’s exact test was used for categorical variables and Mann-Whitney U test for continuous variables (skewed data, two groups). To make sure the results were robust to a potential clustering effect caused by some individuals contributing more than one tooth, Generalized Estimating Equations (GEEs) with the individual as the clustering effect were used. When comparing the two groups, the independent variables were tenderness to percussion, tenderness to apical palpation, positive answers(s) to Q1 or Q2, self-reported chronic pain and sex. For interobserver agreement, kappa statistics were used. κ ≤ 20 is considered to equal slight agreement, 0.21–0.40 fair, 0.41–0.60 moderate, 0.61–0.80 substantial and 0.81–1.0 almost perfect agreement [40]. Since missing data was excluded for individual variables, the valid number of included answers varied among variables. All significance tests were two-sided and conducted at the 0.05 level of significance.

## 3. Results

In the original sample, 550 patients with 1256 root-filled teeth agreed to participate. All participants reporting pain or discomfort from ≥1 root-filled tooth were included in the present analyses comparing pain groups, after exclusion 49 participants with 53 painful root-filled teeth were included in the study. Fig. 1 describes the flow of the included and excluded participants.

**Fig. 1.** Flow chart of inclusion and exclusion of participants. DD: dental disease.

### 3.1 Pain characteristics

Regarding frequency, intensity, duration, quality and provoking factors of pain or discomfort, the two groups were very similar. The findings are summarized below and details are found in Table 1. Pain intensity was significantly higher in DD− group; mean value 3.1 (range 0–7) compared to DD+ group with mean value 1.6 (range 0–6) (*p *= 0.030). The distribution of pain intensity ratings and ranges is found in **Supplementary Figs. 1,2**. For both groups, the most commonly reported pain or discomfort frequency was recurrent pain, selected by 48% of the DD+ group and 43.5% of the DD− group (*p *= 0.687). The pain or discomfort had lasted on average for 24.7 months for the DD+ group and 38.2 months for the DD− group (*p* = 0.476). The pain duration since onset can be expressed differently; 40.0% (n = 12) of the DD+ group and 52.2% (n = 12) of the DD− group reported a duration of >2 years (*p* = 0.38). In the DD+ group, the pain or discomfort was reported to start only on provocation by 62.5% of the patients (n = 15) and spontaneously by 37.5% (n = 9) while in the DD− group it started spontaneously in 56.5% of the patients (n = 13) and only on provocation in 43.5% (n = 10). However, no significant differences in distribution were found between the two groups, *p* = 0.191.

**Table 1.** Comparison of pain characteristics between DD+ and DD− groups. Significant differences (*p *< 0.05) are marked in bold type.

		DD+ group (n = 19–27)	DD− group (n = 16–23)	*p*	Missing data (n)
Tenderness to percussion n (%)		13 (43.3)	7 (30.4)	0.337^a^	0
Tenderness to apical palpation n (%)		8 (26.7)	0 (0)	0.007^c^	0
Swelling n (%)		8 (26.7)	0 (0)	N/A	0
Sinus tract n (%)		3 (10)	0 (0)	N/A	0
Probing depth ≥6 mm n (%)		9 (30)	0 (0)	N/A	1
Apical radiolucency n (%)		21 (70)	0 (0)	N/A	0
Pain frequency n (%)					
	Continuous	4 (16)	6 (26.1)	0.687^a^	5
	Recurrent	12 (48)	10 (43.5)		
	Occasional	9 (36)	7 (30.4)		
Months since pain debut mean (SD)		24.7 (35.6)	38.2 (50.3)	0.476^b^	6
Present pain^d,f^ mean (SD)		1.4 (1.9)	1.5 (2.2)	0.841^b^	3
Worst pain^d,f^ mean (SD)		2.5 (2.2)	3.8 (2.9)	0.116^b^	10
Average pain^d,f^ mean (SD)		1.6 (1.7)	3.1 (2.3)	0.030^b^	10
Pain starts					
	Spontaneously n (%)	9 (37.5)	13 (56.5)	0.191^a^	6
	Only on provocation n (%)	15 (62.5)	10 (43.5)		
Days with pain^f^ mean (SD)		8.6 (11)	11.8 (13.2)	0.380^b^	10
Days abstaining from usual activities^f^ mean (SD)		0 (0)	0 (0)	1.000^b^	10
Pain-related limitation^f^ on:					
	Daily activities n (%)	5 (1)	0 (0)	0.374^b^	10
	Recreational activities n (%)	5 (1)	0 (0)	0.374^b^	10
	Ability to work n (%)	5 (1)	0 (0)	0.374^b^	10
Chronic Pain Grade					
	Low pain intensity, none to low disability Grade I n (%)	19 (90.5)	16 (88.9)	0.505^a^	10
	High pain intensity, without disability Grade IIa n (%)	1 (4.8)	2 (11.1)		
	High pain intensity, low disability Grade IIb n (%)	1 (4.8)	0 (0)		
Analgesics^e^ n (%)		2 (8.3)	2 (10.5)	1.000^c^	10
Antibiotics^e^ n (%)		0 (0)	0 (0)	-	10
Herbal medicine^e^ n (%)		0 (0)	0 (0)	-	10

*^a^: Chi-square test; ^b^: Independent samples Mann-Whitney U Test; ^c^: Fisher’s Exact Test; ^d^: 0–10 numeric rating scale; ^e^: Intake during the past month, because of the tooth pain; ^f^: During the past month. DD: dental disease; SD: standard deviation.*

When the participants described the quality of their pain, the most common descriptors for the DD+ group was tender (n = 13, 48.1%), aching (n = 6, 21.4%), splitting (n = 5, 18.5%) and throbbing pain (n = 4, 14.8%), respectively. The most common descriptors for the DD− group was tender (n = 14, 60.9%), aching (n = 43.5%), heavy (n = 4, 17.4%) and throbbing pain (n = 4, 17.4%). No significant between-group differences were found in the choice of descriptors. **Supplementary Table 1** shows the distribution of all verbal pain quality descriptors.

Some teeth in the DD+ group had multiple clinical and radiographic signs. The distribution is found in Table 2.

**Table 2.** Distribution of clinical and radiographic sign(s) of dental disease within the DD+ group. Data on tooth level. Some combinations occurred in pairs and some in triplets, hence the total numbers do not add up. In the DD− group all signs of dental disease were absent.

	Swelling n (%)	Sinus tract n (%)	Probing depth ≥6 mm n (%)^a^	Apical radiolucency n (%)
Swelling n (%)	N/A	2	4	2
Sinus tract n (%)	2	N/A	1	2
Probing depth ≥6 mm n (%)^a^	4	1	N/A	3
Apical radiolucency n (%)	2	2	3	N/A

*^a^: Missing data n = 1.*

### 3.2 Clinical pain provocation tests

Table 1 shows clinical provocation test data. Tenderness to apical palpation was not reported by anyone in the DD− group, and was thus more common in the DD+ group (*p* = 0.007), while tenderness to percussion did not differ between the groups (*p* = 0.337).

### 3.3 Persistent tooth pain

Persistent tooth pain, defined as pain for at least 8 hours per day and at least 15 days per month the past 3 months was reported by seven participants with seven painful root-filled teeth in the DD+ group and by five participants with five painful root-filled teeth in the DD− group. The details are found in Table 3.

**Table 3.** Demographic and pain characteristics for participants with persistent tooth pain with (DD+) and without (DD−) signs of dental disease.

		All cases	DD+	DD−
Participants; n		12	7	5
Teeth; n		12	7	5
Prevalence				
	Individual level; %^a^	2.2	1.3	0.9
	Tooth level; %^b^	0.96	0.56	0.4
Sex				
	Female; n (%)	7 (58.3)	3 (42.9)	4 (80)
	Male; n (%)	5 (41.7)	4 (57.1)	1 (20)
Age; mean (range)		59.4 (41–74)	57.7 (41–74)	61.8 (52–71)
Average pain intensity^c^; mean (SD)		2.2 (2.1)^d^	2.7 (2.3)^e^	1.5 (1.7)^e^
Worst pain intensity^c^; mean (SD)		2.5 (2.1)^d^	3.0 (2.4)^e^	1.75 (1.7)^e^
Months since pain debut; mean (range)		32.2 (0.7–144)	40.7 (0.7–143.3)	20.4 (6–48)

*^a^: All = 550; ^b^: All = 1256; ^c^: 0–10 numeric rating scale; ^d^: missing data n = 2; ^e^: missing data n = 1. DD: dental disease; SD: standard deviation.*

### 3.4 The impact of pain

The impact of pain on daily activities, expressed as Chronic Pain Grade and as intake of medications, showed that on average, the impact of pain was low with no differences between groups (Table 1).

### 3.5 Patient and clinical characteristics

Table 4 presents the demographics and patient-related and tooth-related characteristics. No significant differences were found between the two groups regarding sex (*p* = 0.961), age (*p* = 0.540) or general health, (*p* = 0.740). Furthermore, no differences could be seen between number of included teeth per participant (*p *= 0.617), jaw position (*p *= 0.431) or tooth type (*p *= 0.752).

**Table 4.** Demographic, patient-related and tooth-related data for all cases together and both pain groups.

Individuals		All cases n = 49	DD+ group n = 27	DD− group n = 22	*p*
Age; mean (SD)		56.6 (13.3)	57.6 (13.5)	55.4 (13.4)	0.540^a^
Age; median (range)		59.0 (21–82)	62.0 (29–82)	58.5 (21–72)	
Sex					
	Female; n (%)	31 (63.3)	17 (63.0)	14 (63.6)	
	Male; n (%)	18 (36.7)	10 (37.0)	8 (36.4)	0.961^b^
≥1 general health concern; n (%)		21 (42.9)	11 (40.7)	10 (45.5)	0.740^b^
Q1 or Q2 3Q/TMD^d,e^ n (%)			7 (26.9)^f^	6 (27.3)	0.978^a^
Chronic bodily pains;^d^ n (%)			11 (40.7)	9 (40.9)	0.990^a^
Root-filled teeth per individual; n (%)					
	1	45 (91.8)	24 (88.9)	21 (95.5)	0.617^c^
	2	4 (8.2)	3 (11.1)	1 (4.5)	
Teeth		n = 53	n = 30	n = 23	
Tooth position					
	Maxilla; n (%)	29 (54.7)	15 (50.0)	14 (60.9)	0.431^b^
	Mandible; n (%)	24 (45.3)	15 (50.0)	9 (39.1)	
Tooth type					
	Incisor/canine; n (%)	12 (22.6)	6 (20.0)	6 (26.1)	0.752^b^
	Premolar; n (%)	8 (15.1)	4 (13.3)	4 (17.4)	
	Molar; n (%)	33 (62.3)	20 (66.7)	13 (56.5)	

*^a^: Independent Samples Mann-Whitney U Test; ^b^: Pearson chi square; ^c^: Fisher’s Exact Test; ^d^: Individual level; ^e^: 3Q/TMD are screening questions for TMD; ^f^: Missing data n = 1. DD+: teeth with signs of dental disease; DD−: teeth without signs of dental disease; SD: standard deviation.*

### 3.6 General health and chronic bodily or orofacial pain

There were no between-group differences for any of the variables. In the DD+ group 40.7% (n = 11) and in the DD− group 45.5% (n = 10) reported at least one general health concern (*p* = 0.740).

In the DD+ group 26.9% (n = 7) and 27.3% (n = 6) in the DD− group gave a positive answer to at least one of the two pain-related questions of the 3Q/TMD; Q1 or Q2 (*p* = 0.978).

Eleven of the participants (40.7%) in the DD+ group and nine of the participants (40.9%) in the DD− group experienced pain from ≥1 body site (*p *= 0.990).

### 3.7 Comparison between the groups—multivariate analyses

Univariate analyses do not take into consideration a possible clustering effect due to some participants contributing with more than one tooth. A multivariate analysis with GEE was therefore performed to confirm the robustness of the findings. This analysis found no statistically significant effects (ORs) (**Supplementary Table 2**, *p* = 0.33–0.86), indicating absence of a clustering effect. The variable “tenderness to apical palpation” was excluded from the analysis since no participant in DD− reported this.

### 3.8 Prevalence of odontogenic and non-odontogenic pain or discomfort associated with root-filled teeth

The original cross-sectional study involved 550 patients with 1256 root-filled teeth. The number of patients who reported a painful tooth was 53 (intention to include) rendering a pain prevalence of 9.6%. However, data was missing for four patients, thus 49 patients with 53 teeth were included in the analyses (Fig. 1); giving a prevalence of 8.9%. Twenty-two of the 550 originally included participants were assigned to the DD− group, which gives a 4.0% prevalence of pain or discomfort from root-filled teeth without signs of dental disease on individual level, and on tooth level a prevalence of 1.8% (23/1256 root-filled teeth). Twenty-seven of the participants were assigned to the DD+ group, which gives a 4.9% prevalence of pain or discomfort from teeth with signs of dental disease on individual level, and a prevalence of 2.4% on tooth level (30/1256 root-filled teeth).

### 3.9 Observer agreement of radiographic assessment

In the radiographic assessment all observers agreed on presence of apical radiolucency in 13 teeth (24.5%), two observers agreed on apical radiolucency in 8 teeth (15.1%) and only one observer noted apical radiolucency in 6 additional teeth (11.3%). All three observers agreed on absence of apical radiolucency in 26 teeth (49.1%), giving a total 3-observer agreement rate of 73.6%, and ≥2-observer agreement rate of 88.7%. κ values for the interobserver agreement ranged 0.525–0.755, which equals moderate to substantial agreement [40]. The details can be found in **Supplementary Table 3**.

## 4. Discussion

The most important finding of this study was that there were very few differences in clinical as well as patient-related characteristics between those experiencing pain or discomfort in root-filled teeth due to a clear dental inflammatory disease and those with no obvious signs of odontogenic etiology, highlighting the challenges in correctly diagnosing painful root-filled teeth. This new knowledge should conceivably change the clinician’s perspective when assessing a tooth and diagnosing their patient, as well as reject an uncritical understanding of tooth pain as “always” being a representation of local inflammation and therefore amenable to dental treatment.

The lack of clear differences between the groups regarding pain characteristics, impact of pain, and background factors can have several reasons. One reason might be that the measuring methods of this study were not able to capture differences between pain origins or were not sensitive enough to capture small differences. Since this was a practice-based study, the examinations corresponded to routine clinical protocols in general practice, which are normally considered appropriate for diagnosis, and the patient questionnaires used are well-known and validated. Another possible reason might be that the pain groups were assigned based on incorrect assumptions, *e.g.*, that more patients in fact had inflammatory dental disease that went undetected and therefore cases were misclassified; but as discussed below, the group definitions were conservative. A third possible reason would be that the two groups in fact have very similar clinical presentations, and given the wide variation in pain intensity, frequency, and verbal descriptions revealed, we believe this explanation has the strongest support in data. A fourth reason might be that some participants in the DD+ group in fact had asymptomatic apical pathosis and coexisting TMD pain referring to the tooth, as was reported in a recent study to occur in 8% of the endodontic patients seeking RCT due to tooth pain [41]. The site of pain would then be the tooth, but the true source would be non-odontogenic. TMD pain in patients has been reported to refer to tooth-bearing regions in about 40% of cases [42], which suggests that there is a need for dentists to exclude a myofascial source of tooth pain, especially in cases when other clinical signs clearly indicating an odontogenic pain origin are lacking.

### 4.1 Participants, group assignments and quality of data

The patients in this study were adult individuals who were scheduled for a regular check-up and did not seek care due to a problem, *e.g.*, tooth pain, which would suggest that they may be of pain or discomfort from a root-filled tooth. In contrast, the majority of patients referred to a specialist clinic for a root-filled tooth had more pronounced symptoms such as pain and swelling [43].

Systematic misclassification of cases could be one explanation of the lack of identified differences. The DD+ and DD− groups were defined using a complex standard including both clinical and radiographic parameters, a strategy often reported to increase the diagnostic certainty, *e.g.*, for TMD diagnoses [9, 44]. The criteria categorized the tooth as DD+ if any sign of dental disease (clinical and/or radiographic) was present. The sensitivity of the radiographic examination was increased by using more than one periapical intraoral radiograph per tooth [45]. Compared to periapical and panoramic images, Cone Beam Computed Tomography (CBCT) was reported to have higher sensitivity to identify apical bone destruction [46, 47], and yield a larger number of apical radiolucencies and other important features such as missed, c-shaped, or over-/underfilled root canals and vertical bone defects [10, 48, 49, 50]. It is possible that CBCT would have identified a larger proportion of the cases as DD+. However, since data were collected in general practice under standard routine check-up circumstances, this was not feasible. In addition, when CBCT imaging was compared to histopathologic examination, the positive predictive value of CBCT to identify AP was considerably lower for root-filled compared to non-root-filled teeth, in the range 0.48–0.64 [47]. Thus, it is conceivable that using CBCT would have overestimated the prevalence of AP; *i.e.*, identified teeth without periapical disease as diseased.

In addition, since the time of RCT was not known in this study, it is unknown if a radiolucency observed represented an inflammatory state (AP) or healing in progress. If apical radiolucency is noted in combination with symptoms or abnormal clinical findings, it is more likely to represent active disease, such as apical periodontitis, marginal periodontitis [7], or vertical root fracture [51], especially in root-filled teeth which lack a physiological barrier against infection of the pulp space, generally have less remaining tooth structure and/or are heavily restored. In summary, our approach to group assignment was inclusive for DD+, increasing the chance of “true positives” but at the same time the risk of “false positives”. A more restrictive approach to inclusion in DD− group required absence of all clinical and/or radiographic signs of disease. Therefore, our approach is more likely to have misclassified cases as DD+ than the opposite.

Since the inclusion criteria was “pain during the last 3 months”, there is a theoretical risk of having included participants who had a painful tooth during the past three months but received RCT for it, after which the pain subsided. The risk of this is very low since the screening process took place some months before the data collection and only patients who already had root-filled teeth at the time of the pre-screening of the records were asked for participation.

The quality of clinical data may also affect the group assignment. Since this was a practice-based study, a possible limitation is that the dentists providing data were not calibrated in the clinical examination technique. To limit bias due to differences in procedures, the study coordinator (JJS) visited all clinics prior to the data collection to clarify how the clinical protocol should be interpreted.

To identify group differences, the sample size needs to be adequate. This study included 49 participants with 53 painful root-filled teeth, which surpassed the estimated sample size needed for the main outcome measure pain intensity. Several other studies have compared the characteristics of odontogenic and/or non-odontogenic pain (Atypical Odontalgia (AO), TMD) with similar sample sizes or smaller [52, 53, 54] whereas only few have reported on larger samples [55].

Data was missing for some teeth, n = 3–10; 5.7–18.9%. The variables with missing values were quality of the pain n = 3, present pain intensity n = 3, frequency n = 5, duration since onset n = 6, provoking factors n = 6, worst pain intensity n = 10, average pain intensity n = 10, days with pain past month n = 10, pain-related limitation n = 10, intake of analgesics, antibiotics and herbal remedies n = 10. No imputation was made for missing data in the statistical analyses.

### 4.2 Pain characteristics

On average, the pain characteristics were quite similar between the groups. The majority had mild pain, but the average intensity was higher in the DD− group. Similar relationships were reported in one study comparing odontogenic and non-odontogenic pain 6 months after RCT [10], and in another study comparing root-filled teeth with complete *vs.* incomplete healing 5–14 months post-RCT [5]. In all three studies, patients without clear signs of dental disease reported higher pain intensity than patients with dental inflammatory disease likely to be their pain cause. The potential implication is that with a higher pain intensity the patients are more prone to seek treatment [56] and the dentists could experience a pressure to initiate treatment [57], suggesting a risk of unnecessary endodontic retreatment being performed in this group.

Both groups had similar distribution of pain frequency; in both groups “recurrent pain” was reported by almost half of the participants. The duration since its onset was long for both groups, DD+ >2 years *vs.* DD− >3 years. Similarly, Daline and co-workers reported that in about one fifth of their patients, the pain continued and was still present 3.4 years post-RCT [58]. Recall bias is always a possible risk when working with data remembered in retrospect [59]. This means that, *e.g.*, the reported duration of pain since the onset might not be exact. But overall, we estimate that the pain duration was likely to be long in both groups; roughly 4/10 in the DD+ group and half of the DD− group reported a pain duration since onset of >2 years.

There were also no differences between groups regarding what triggered the isolated pain episodes, although the patients in the DD+ group tended to report that it started more often on provocation rather than appearing spontaneously while the DD− group reported the opposite. In the clinical assessment, pain on palpation of the apical area was reported by about a quarter of the individuals in the DD+ group, and not by any participant in the DD− group. The latter conflicts with another study in which 38% of patients with non-odontogenic persistent pain after RCT reported pain on apical palpation [10].

Tenderness to percussion was equally frequent in both groups. Both parameters represent a state of allodynia, usually attributed to central sensitization, either induced by locally released inflammatory mediators sensitizing peripheral nociceptors [60] as in AP [61], or the sensitization of second-order neurons by ongoing and intense afferent input leading to increased processing of the signals in the higher brain centers [62, 63]. Patients with persistent tooth pain are known to exhibit tenderness to percussion [64], experienced by a majority of patients with tooth pain of odontogenic as well as non-odontogenic origin [10]. It has previously been suggested that spontaneous pain is a clinical feature of neuropathic pain [65] while pain on provocation would indicate inflammatory tooth pain associated with a specific tooth [62].

### 4.3 Prevalence of painful root-filled teeth

The participants in this study were drawn from a larger sample of patients with root-filled teeth presenting for their annual check-up. In the larger sample of 550 patients with 1256 root-filled teeth, the prevalence of odontogenic tooth pain or discomfort (represented by the DD+ group) was 4.9% on an individual level and 2.5% on tooth level. Our post-analysis of published data from 10 retrospective studies and one prospective study [4, 8] estimated the prevalence of odontogenic pain ≥6 months after root canal treatment roughly to 2–3% (on tooth level), which is consistent with our findings.

Correspondingly, the prevalence of pain or discomfort from teeth without signs of inflammatory dental disease was found to be 4.0% on the individual level, in agreement with a meta-analysis of 10 studies reporting a pooled prevalence of non-odontogenic tooth pain 1–6 years after RCT to be 3.4% [8], and a more recent retrospective practice-based study reporting 3.1% prevalence of non-odontogenic tooth pain 3–5 years post-RCT [4].

### 4.4 Persistent tooth pain

Persistent pain has been reported to affect root-filled teeth, and as mentioned above, several pain origins are possible. In the current study, the specific pain origin for each case in the DD− group was not possible to establish. Nevertheless, it was of interest to examine the prevalence of persistent pain on the population level. Five participants with five root-filled teeth (a prevalence of 0.9% on the individual level and 0.4% on tooth level) fulfilled the temporal criteria for persistent dento-alveolar pain disorder (PDAP), “pain more than 8 hours a day for at least 15 days per month the last three months” [28]. In contrast, another study establishing pain diagnoses >6 months after RCT reported that 2/19 participants (10.5%) of participants with persistent pain were diagnosed with PDAP [10]. The large difference is likely a reflection of participant selection and study design; the present cross-sectional study examined an adult population visiting their dentist for a check-up, while the Nixdorf *et al*. [10] (2015) study was a prospective study of RCT. It must be emphasized that PDAP is a diagnosis of exclusion, appropriate only when all other possible pain origins (*e.g.*, TMD, sinusitis, neuropathic pain, headache) have been excluded with reasonable certainty, which was not done in this study.

Our findings underscore that persistent pain is not always a reason to seek care. The intensity may be mild and might therefore be neglected, ignored or missed by patients and dentists, possibly due to a lack of understanding of its cause. Future research could focus on identifying reasons for not addressing pain in a root-filled tooth. Discomfort from root-filled teeth may not always be identified as pain. Eleven of the participants answered YES to the question “Have you experienced pain or discomfort (swelling, tenderness on chewing or any other problem) from any of your root-filled teeth at any time during the last 3 months?” but still rated their average pain intensity as zero. Similarly, 29 out of 264 participants (11%) in a prospective study rated their pain intensity as zero but also described the quality of their symptoms according to the McGill Pain questionnaire [5]. One possible interpretation of this is that they experience dysesthesia rather than pain. The definition of dysesthesia according to IASP is “an unpleasant abnormal sensation, whether spontaneous or evoked” [66]. It is often associated with damage to the peripheral sensory nerves, including the trigeminal nerve [21], and reported iatrogenic reasons include endodontic treatment and local anesthesia [37]. In the present study no differences were seen between the two groups concerning the SF-MPQ, which concurs with other studies exploring the diagnostic value of pain quality to distinguish between pain conditions [52, 67]. Based on descriptive words from patients, a fourteen-item screener was developed with the purpose to differentiate AO and PDAP patients from other oral pain conditions. The reported sensitivity was 0.77 and specificity was 0.69, suggesting that the screener could be used with caution together with standard clinical examination [68].

### 4.5 The impact of pain

On average, the impact on life was very low. Despite long pain duration since onset in both groups, the participants reported little effect on daily activities (GCPS grades I–II with either low or high pain intensity) and very few took analgesics for the pain. A study evaluating pain from root-filled teeth 6 months after RCT reported similar impact but a larger proportion (42.6%) of participants took pain medications [2] which may reflect a shorter pain duration since onset and thus possibly still acute pain after RCT. A follow-up study of patients with AO reported more severe impact on life from persistent pain; about 1 in 10 patients originally recruited from orofacial pain clinics were in grade III or IV, and 3/4 in grades I–II after seven years [54]. Again, the differences are likely explained by differences in participant selection.

### 4.6 Patient characteristics: General health, TMD and other chronic pain

In patients with chronic pain compared to patients without chronic pain, increased odds ratios have been reported for general health issues such as diabetes, hypertension, ischemic heart disease, heart failure, acute myocardial infarction, parkinsonism and mood/anxiety disorders in patients [69]. This may conceivably be associated with systemic factors, but the direction of a possible causal relationship remains unclear. This study found no difference between the two pain groups regarding presence of general health problems. A retrospective cross-sectional study from specialist care similarly reported no group differences regarding presence of “other health comorbidities” between five different orofacial pain diagnoses [70], suggesting that general health concerns are similar between pain conditions.

TMD pain is a frequent finding in the adult population in epidemiological studies, (5–12%) [71]. In the current practice-based study, a complete examination of the masticatory system such as the DC/TMD [9] was not performed, so no TMD pain diagnoses, *e.g.*, myofascial pain or arthralgia were made. However, the 3Q/TMD screening instrument was validated in its entirety and has shown good validity with sensitivity and specificity >0.8 [25]. We did not analyze the third question unrelated to pain, and therefore identification of all TMDs is less certain, but positive responses to the pain-related questions 1 and 2 are still likely to reflect the occurence of painful TMD specifically. The estimated prevalence was about one in four participants, with no difference between the two pain groups. Other studies have reported an association between pain from root-filled teeth and TMD [1, 5, 10]. In a study of chronic tooth pain and TMD, half of the patients with painful teeth also had myofascial TMD [52]. A study on presence of TMD among endodontic patients reported a 54% prevalence of painful TMDs [41]. The specific pathophysiologic mechanisms of myofascial pain are unknown, but a theory is that sustained contraction of the muscles may be a factor, and that persistent and annoying pain *e.g.*, toothache can produce a local muscle response, triggering muscle pain [72, 73].

Chronic Overlapping Pain Conditions (COPCs) are pain conditions that frequently occur together; some examples are fibromyalgia, irritable bowel syndrome, TMD, lower back pain and migraine [74] and possibly also PDAP [75]. Among the COPCs, pain amplification and an abnormal sensitivity to pain is a common feature [74]. This begs the question: do patients with pain from their root-filled tooth also have other bodily pains, and does it differ between inflammatory dental pain origin and non-odontogenic pain origin? In this study, no association could be seen between long-lasting pains in the rest of the body and tooth pain origin; the prevalence was similar in both groups. Similarly, a study comparing patient questionnaire data 3 years post-RCT performed by mainly general dentists in a practice-based study found no differences [58].

## 5. Conclusions

The pain characteristics, such as intensity, frequency, duration since onset, quality, and provoking factors, as well as dental examination findings were similar in patients experiencing pain from root-filled teeth with and without signs of inflammatory dental disease. The exceptions were higher pain intensity and absence of tenderness to apical palpation in those without signs of dental disease. The everyday impact of the pain was equally minimal regardless of the assumed pain origin. The patient characteristics recorded in this study, such as general health and presence of chronic bodily or orofacial pain, affected both groups alike and therefore could not help explain the pain on group level. The similarities in clinical presentation of the two groups underscore the difficulties in correctly diagnosing painful conditions of root-filled teeth.

## 6. Clinical implications

When a patient with a root-filled tooth exhibits pain or discomfort from the tooth, one should be careful when establishing a diagnosis. Unless clear signs of inflammatory dental disease are identified, it is quite possible that the pain has a non-odontogenic origin. Symptoms and signs of tooth pain of different origins may be very similar and may not be sufficient to make a clear differential diagnosis. The findings of this study suggest that routine methods using brief pain history and a focused dental examination are insufficient to safely distinguish between pain origins. Instead, additional examination or tools need to be used or developed to help discriminate between a local inflammatory pain and various non-odontogenic sources of pain perceived as emanating from a root-filled tooth.

## Supplementary Material


