# Supplementary material for: Characteristics and impact of pain from root-filled teeth. A practice-based cross-sectional study comparing painful teeth with and without signs of inflammatory dental disease
Source: J Oral Facial Pain Headache. 2024 Mar 12;38(1):64–76. doi: 10.22514/jofph.2024.007 (PMC11774281; doi:10.22514/jofph.2024.007)
Supplement: Supplementary file 1 [file Supplementary-material.docx]

Supplementary material

Supplementary Fig. 1. The distribution of average pain intensity (0–10 Numeric rating scale) during the past month for the two groups, DD+ and DD−. Missing data; DD− n = 4, DD+ n = 6. DD+: teeth with signs of dental disease; DD−: teeth without signs of dental disease. Data on tooth level.

Supplementary Fig. 2. The distribution of worst pain intensity (0–10 Numeric rating scale) during the past month for the two groups, DD+ and DD−. Missing data; DD− n = 4, DD+ n = 6. DD+: teeth with signs of dental disease; DD−: teeth without signs of dental disease. Data on tooth level.

Supplementary Table 1. Pain descriptors for the two pain groups using the Short-Form McGill Pain Questionnaire (SF-MPQ). All positive response alternatives “mild”, “moderate” and “severe” were combined and contrasted with the negative response “none”.

|  | DD+ group^a^  n = 27 | DD− group n = 23 | *p* |
| --- | --- | --- | --- |
| Throbbing n (%) | 4 (14.8) | 4 (17.4) | 1.000^b^ |
| Shooting n (%) | 0 (0) | 2 (8.7) | 0.207^b^ |
| Stabbing n (%) | 0 (0) | 2 (8.7) | 0.207^b^ |
| Sharp n (%) | 1 (3.7) | 1 (4.3) | 1.000^b^ |
| Cramping n (%) | 0 (0) | 1 (4.3) | 0.460^b^ |
| Gnawing n (%) | 0 (0) | 0 (0) | - |
| Hot/Burning n (%) | 0 (0) | 0 (0) | - |
| Aching n (%) | 6 (21.4) | 10 (43.5) | 0.091^c^ |
| Heavy n (%) | 1 (3.7) | 4 (17.4) | 0.167^b^ |
| Tender n (%) | 13 (48.1) | 14 (60.9) | 0.368^c^ |
| Splitting n (%) | 5 (18.5) | 2 (8.7) | 0.430^b^ |
| Tiring/Exhausting n (%) | 2 (7.4) | 0 (0) | 0.493^b^ |
| Sickening n (%) | 0 (0) | 0 (0) | - |
| Fearful n (%) | 1 (3.7) | 0 (0) | 1.000^b^ |
| Punishing/Cruel n (%) | 1 (3.7) | 0 (0) | 1.000^b^ |

^a^: Missing data n = 3^;^ ^b^: Fisher´s exact test^;^ ^c^: Pearsons chi-square test.

DD+: teeth with signs of dental disease; DD−: teeth without signs of dental disease.

Supplementary Table 2. Multivariate analysis (Generalized Estimating Equation, GEE) to reveal a potential clustering effect by the individual on the association between pain in a root-filled tooth (dependent variable) and patient characteristics (independent variables) in individuals contributing >1 root-filled painful tooth.

|  | OR | 95% CI | *p* |
| --- | --- | --- | --- |
| Tenderness to percussion | 1.84 | 0.53–6.39 | 0.33 |
| Positive answer to 3Q/TMD Q1 and/or Q2^a^ | 0.80 | 0.21–3.07 | 0.75 |
| Chronic pains | 1.56 | 0.43–5.68 | 0.50 |
| Sex |  |  |  |
| Male (reference) | 1 |  |  |
| Female | 0.89 | 0.24–3.25 | 0.86 |

Missing data n = 1.

^a^: Screening questions for TMD.

Odds ratios (OR) and 95% confidence intervals (CI) for belonging to the DD+ group, with DD− group as the reference. *p* < 0.05 was considered statistically significant.

Supplementary Table 3. Distribution of observer agreement for radiographic assessment of the painful teeth. Normal periapical condition and widened periodontal ligament space was merged into one group. If ≥2 observers agreed on presence of apical radiolucency the tooth was noted as having apical radiolucency.

|  | N^a^ (%) |
| --- | --- |
| All three observers agreed on presence of apical radiolucency | 13 (24.5) |
| Two observers: presence of apical radiolucency | 8 (15.1) |
| One observer: presence of apical radiolucency | 6 (11.3) |
| All three observers agreed on absence of apical radiolucency | 26 (49.1) |

^a^: n = 53.
